# Unusual usage of the automated stapler in gynecologic oncology: method for diaphragmatic full thickness implant resection without entrance to pleural space

**DOI:** 10.4274/jtgga.galenos.2020.2020.0008

**Published:** 2020-12-04

**Authors:** Günsu Kimyon Cömert, Alper Karalok, Ciğdem Kılıç, Derman Başaran, Fatih Kılıç, Osman Türkmen, Taner Turan

**Affiliations:** 1 Clinic of Obstetrics and Gynecology, University of Health Sciences Turkey, Ankara Etlik Zübeyde Hanım Women’s Health Training and Research Hospital, Ankara, Turkey

 To the Editor,

The improved survival impact of achieving residue zero cytoreduction were proven in many studies for ovarian carcinoma (1,2). In those, one of the most commonly involved sites is the diaphragm in up to 40% of cases. Diaphragmatic involvement is one of the most common reasons for the failure to achieve complete or optimal cytoreduction surgery (3). Although there has been improvements in technique, experience, and education over the years, there are still concerns about complications and management of diaphragmatic tumor resection, especially in the presence of full thickness implants.

Therefore, we would like to describe a technique for resection of diaphragm full thickness implants without entrance to the pleural space. Firstly, the liver was mobilized. Diaphragm striping was performed up to the full thickness implant. When an unresectable area was reached, the following technique was performed to resect the full thickness implant. The steps of the technique were: (i) after identification of the full thickness implant borderlines, sutures were placed to the medial, middle and lateral edges of the full thickness implant to more easily perform traction (Figure 1); (ii) an automated stapling device, such as thoraco-abdominal stapler DST series^TM^ (Figure 2) or gastro-intestinal anastomosis stapler (DST series^TM^) were placed transversally to diaphragm, under the full thickness implant which had been displaced by traction; (iii) in order to avoid lung parenchymal injury, the ventilator was temporarily turned off after exhalation, while the stapler was locked up; (iv) the stapler was locked up to place the sutures automatically; (v) the full thickness implant above the staplers was resected via manual scalpel for thoraco-abdominal stapler or by the integrated automated scalpel for gastro-intestinal anastomosis stapler, and the stapler was opened; (vi) the resection was completed without entrance to pleural space; and (vii) final control for air leakage using a bubble test, was performed. There were either no, or minimal, asymptomatic pleural effusion, and no pneumothorax. There was also no need for thoracentesis during the postoperative period in both patients in our institution. Diaphragmatic muscle invasion of a high-grade, serous ovarian carcinoma was reported in pathologic results [institutional review board (approval number: 07/2019/90057706-799)].

One of the concerns when undertaking diaphragmatic full thickness implant resection is pulmonary complication. Additionally, entrance to the pleural cavity increases the possibility of prophylactic chest tube application (4,5). The technique described here may have an advantage in minimizing the occurrence of pneumothorax and the amount of pleural effusion by avoiding pleural entrance. Thus, this may encourage less use of a prophylactic chest tube, decrease the need for thoracentesis, and also lessen postoperative morbidity. Our technique may also make diaphragmatic full thickness implant resection viable as part of minimally invasive surgeries. The undeniable fact that the operation time is longer in the presence of diaphragmatic full thickness implant resection in contrast to striping, because of the need for manual closure by suture. This technique may have an additional advantage in decreasing operation time because of automatically suturing. Kapnick et al. (6) showed that the probability of pleural/parenchymal involvement was higher in the presence of more than 5 cm full thickness implant. Therefore, we believe that this technique may be a good option in <4 cm full thickness implants.

To the best of our knowledge, this is the first report of the usage of a thoraco-abdominal stapler for resection of diaphragmatic full thickness implant without entrance to the pleural space. Diaphragmatic full thickness implant resection with stapler appears to be safe, practical and an easy to learn surgical technique. There is a need for large scale studies to evaluate the conclusions of this technique.

## Figures and Tables

**Figure 1:**
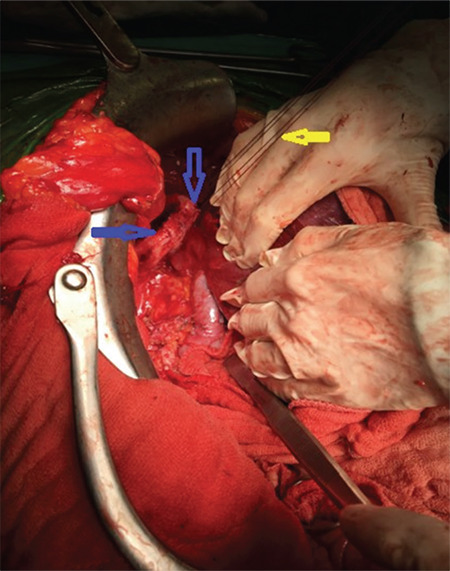
Sutures placed to the medial edge, the middle and the lateral edge of the full thickness implant to perform traction easier

**Figure 2:**
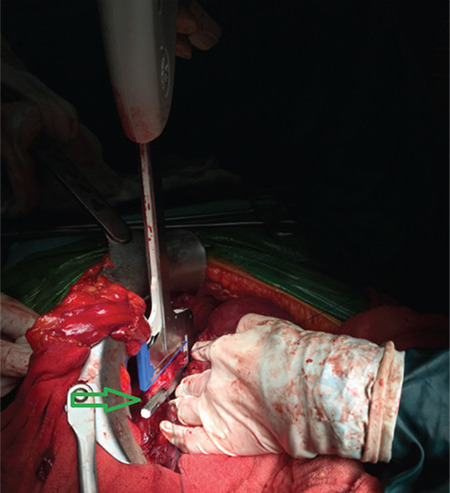
Thoraco-abdominal stapler (DST seriesTM, 30 mm) was placed under the hauled full thickness implant transversally to diaphragm
